# Global landscape and emerging trends in programmable mRNA-based therapeutics for myocardial infarction: a multi-database bibliometric analysis

**DOI:** 10.3389/fmed.2026.1957977

**Published:** 2026-09-28

**Authors:** Ying-liang Zhang, Jia-yi Liu, Yu-chen Meng, Yan-tong Zhou, Jian-zheng Xu, Qi-ling Ma, Yu-ge Chen, Chi Zhang, Jun-chi Zhu, Jian Dong, Chen-hao Zhang, Kai-yan Fan, De-zhu Liu, De-you Jiang

**Affiliations:** 1 Graduate School, Heilongjiang University of Chinese Medicine, Harbin, Heilongjiang, China; 2 Second Clinical Medical College, Heilongjiang University of Chinese Medicine, Harbin, Heilongjiang, China; 3 First Clinical Medical College, Heilongjiang University of Chinese Medicine, Harbin, Heilongjiang, China; 4 School of Basic Medical Sciences, Heilongjiang University of Chinese Medicine, Harbin, Heilongjiang, China

**Keywords:** bibliometric analysis, knowledge mapping, myocardial infarction, programmable mRNA, research trends

## Abstract

**Background:**

Myocardial infarction (MI) remains a leading cause of mortality worldwide. Although current reperfusion-based interventions have significantly improved clinical outcomes, they are insufficient to restore lost cardiomyocytes or repair irreversible myocardial injury. Programmable messenger RNA (mRNA)-based therapeutics have emerged as a cutting-edge strategy for cardiac regenerative repair owing to their advantages of transient and controllable protein expression, high design flexibility, and avoidance of genomic integration. However, the global research landscape, collaborative networks, and evolving scientific priorities in this rapidly expanding field have not yet been systematically characterized.

**Methods:**

A comprehensive bibliometric analysis was conducted using publications retrieved from the Web of Science Core Collection (WoSCC) and PubMed databases from 2012 to 2026. After literature screening and deduplication, 814 publications from WoSCC and 4,155 publications from PubMed were included. VOSviewer, CiteSpace, and the R package Bibliometrix were used to conduct parallel analyses of annual publication trends, country- and institutional-collaboration networks, journal and author distributions, reference citation bursts, and keyword-based thematic evolution.

**Results:**

WoSCC analysis revealed a fluctuating but overall increasing publication trajectory, with annual research output peaking at ∼90 publications in 2023. The evolution of this field could be categorized into three stages: an emerging phase (2012–2016), a developmental phase (2017–2021), and an accelerated expansion phase (2022 onward). China and the United States represented the leading contributors, while institutions including Harvard University and the Chinese Academy of Medical Sciences-Peking Union Medical College served as major research hubs. Circulation Research and Circulation occupied central positions within the co-citation network. ZANG, LICHUN was identified as the most productive author, whereas the landmark 2013 study by Zangi L represented the most influential foundational contribution to the field. Parallel analysis of PubMed further validated the identified evolutionary trends and highlighted angiogenesis as a promising yet underexplored research direction.

**Conclusion:**

Research on programmable mRNA therapeutics for MI is rapidly expanding, with the focus shifting from fundamental molecular mechanisms to translational applications that enable precise inflammatory modulation, fibrosis reversal, and revascularization. Future breakthroughs will likely depend on the development of efficient and cardiac-targeted delivery platforms, as well as deeper exploration of epitranscriptomic therapeutic strategies, to accelerate clinical translation.

## 1 Introduction

Cardiovascular disease (CVD) remains the leading cause of death globally, accounting for approximately 17.9 million deaths per year, representing about 32% of all deaths worldwide (1). Ischemic heart disease (IHD) alone contributed to an estimated 9.4 million deaths in 2021 and has maintained its position as the single largest contributor to cardiovascular mortality for over three decades (2, 3). The socioeconomic burden is equally striking. IHD-related healthcare expenditures and productivity losses exceed 100 billion US dollars annually in high-income countries and continue to rise in low- and middle-income regions where disease incidence is accelerating (4). Myocardial infarction (MI) is the most acute and life-threatening manifestation of IHD (5). Acute coronary artery occlusion triggers irreversible cardiomyocyte death, followed by progressive fibrosis, ventricular remodeling, and a cascade toward heart failure (6). Despite improvements in emergency reperfusion, the long-term mortality rate following MI remains high, with approximately one in six survivors developing heart failure within five years (7, 8).

Percutaneous coronary intervention (PCI) and thrombolytic therapy are the established front-line treatments for acute MI (9). These interventions restore epicardial blood flow and salvage at-risk ischemic myocardium (10). Their benefit, however, is largely confined to restoring macrovascular patency (11). Neither approach replenishes lost cardiomyocytes, halts the fibrotic remodeling that progressively stiffens the ventricular wall, nor rebuilds the microvascular network disrupted during infarction (12). There is therefore a persistent and clinically important gap in the treatment landscape for post-infarction cardiac repair and regeneration.

Viral vectors have been the traditional tool for cardiac gene therapy (13). Their clinical translation, however, has been hindered by several well-documented limitations (14). Genomic integration poses a risk of insertional mutagenesis. Sustained transgene expression can provoke chronic immune responses (15). Systemic biodistribution leads to off-target effects in non-cardiac tissues (16). Non-viral delivery of messenger RNA (mRNA) has gained considerable attention as an alternative strategy (17). Programmable mRNA platforms now include nucleoside-modified mRNA (modRNA), *in vitro*-transcribed mRNA (IVT mRNA), self-amplifying RNA (saRNA), and lipid nanoparticle-encapsulated mRNA (LNP-mRNA) (18). These platforms avoid genomic integration by design (19). They produce transient protein expression lasting several days to a few weeks, which aligns with the temporal window needed for post-infarction repair (20). Engineered nucleosides, optimized 5′ cap structures, and extended poly (A) tails collectively reduce innate immune recognition of the delivered RNA (21). When packaged in targeted lipid nanoparticles, these transcripts can accumulate in the infarcted myocardium (22). Preclinical studies have demonstrated that mRNA encoding vascular endothelial growth factor A (VEGFA) promotes neovascularization, that Yes-associated protein 5SA mutant (YAP5SA) and Follistatin-like 1 (FSTL1) transcripts stimulate cardiomyocyte proliferation, and that combined delivery of anti-apoptotic and anti-fibrotic payloads attenuates scar formation and preserves cardiac function (23, 24).

Interest in programmable mRNA for MI has grown rapidly since 2012, driven by advances in RNA chemistry, nucleoside modification, and targeted nanoparticle design (25). The resulting body of literature now spans molecular biology, nanomedicine, cardiovascular pharmacology, and translational medicine (26). Despite this breadth, no study to date has mapped the global research landscape of this field in a systematic and quantitative manner (27–29). Bibliometric analysis offers a well-established methodology for this purpose. It can identify publication trends, core research groups, influential journals, and evolving thematic directions while minimizing observer bias (30). Such an analysis is particularly valuable in a rapidly expanding and interdisciplinary field where an overview of the research trajectory can help researchers and funding agencies prioritize future directions.

This study aimed to provide a comprehensive bibliometric overview of programmable mRNA research for MI over the 15-year period from 2012 to 2026. We retrieved relevant publications from the Web of Science Core Collection (WoSCC) and PubMed. After standardized PRISMA-based screening and Python-assisted deduplication, 814 peer-reviewed articles from WoSCC and 4,155 from PubMed were included for analysis. We employed VOSviewer, CiteSpace, and the R package Bibliometrix to examine annual publication and citation trends, citation distributions by article type, country and institutional collaboration networks, journal co-citation patterns, author productivity distributions, keyword co-occurrence clusters, burst keyword detection, and thematic evolution maps. The specific objectives were as follows: (1) to characterize the overall publication patterns and output distribution in this field; (2) to identify the core research teams, high-impact journals, and landmark publications; (3) to trace shifts in research hotspots and developmental phases across the 15-year study window; and (4) to discuss existing limitations and translational challenges and to outline future directions for programmable mRNA-based cardiac regeneration. This dual-database bibliometric analysis provides a quantitative reference that may help researchers orient themselves within the evolving landscape of this rapidly growing field.

## 2 Materials and methods

### 2.1 Data sources and search strategy

Two databases were searched in parallel to ensure broad coverage. The Web of Science Core Collection (WoSCC) served as the primary source for bibliometric analysis, as it provides comprehensive citation records, author keywords, Keywords Plus, and cited reference data essential for co-citation and burst detection analyses. PubMed was searched simultaneously as a complementary database to capture publications indexed outside the WoSCC ecosystem. Because PubMed records lack several bibliometric fields present in WoSCC—such as Keywords Plus, cited references, and full author affiliation details—the two datasets were analyzed independently in parallel rather than merged, allowing each database to provide complementary perspectives on the research landscape.

The search covered publications from January 1, 2012, to July 15, 2026. Owing to citation accumulation time and data completeness considerations, the WoSCC-based temporal trend analysis was restricted to the 2012–2023 window. The search string combined three conceptual blocks using Boolean operators: (1) programmable mRNA terms, (2) myocardial infarction and related ischemic cardiac pathology, and (3) therapeutic or regenerative intervention terms. For WoSCC, the query was applied to the Topic field, which searches titles, abstracts, author keywords, and Keywords Plus. For PubMed, a corresponding search was adapted to the Title, Abstract, and MeSH fields. The complete search strings are provided in Supplementary Table S1.

### 2.2 Study selection

Study selection followed the PRISMA 2020 guidelines (31). Two reviewers independently screened titles and abstracts using Rayyan, with disagreements resolved by consensus. Full-text articles were assessed against the following inclusion criteria: (i) original research articles or reviews; (ii) studies investigating programmable mRNA technologies (modRNA, IVT mRNA, saRNA, or LNP-mRNA) applied to myocardial infarction, ischemia-reperfusion injury, or post-infarction cardiac repair; (iii) studies addressing mRNA-based therapeutic mechanisms, delivery systems, or translational applications in the cardiovascular domain; and (iv) articles published in English in peer-reviewed journals.

Exclusion criteria were: (i) editorials, conference abstracts, letters, book chapters, errata, and retractions; (ii) studies focused exclusively on mRNA vaccines for infectious diseases or oncology without cardiovascular relevance; (iii) studies limited to viral vectors, DNA plasmids, or non-coding RNAs without a programmable mRNA component; and (iv) duplicate records across databases. The PRISMA flow diagram is shown in Figure 1.

**Figure 1:**
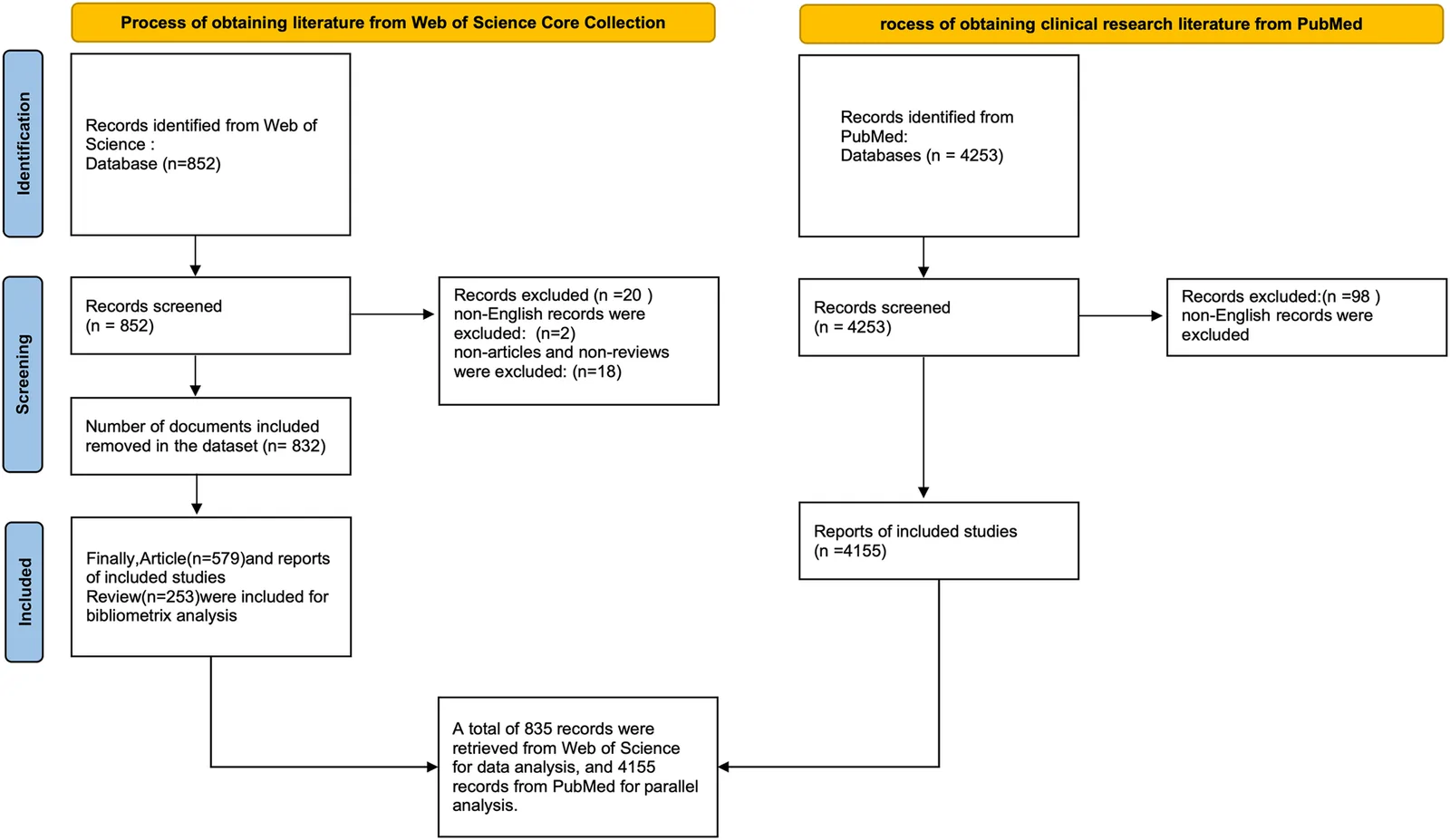
PRISMA-adapted flow diagram of the literature retrieval and screening process.

### 2.3 Data extraction and preprocessing

Bibliometric metadata were extracted into a standardized Microsoft Excel 2024 template. For WoSCC records, fields included title, authors, affiliations, country/region, journal, publication year, document type, citation count, author keywords, Keywords Plus, abstract, and cited references. For PubMed records, available fields included title, authors, journal, publication year, document type, author keywords, MeSH terms, and abstract. PubMed records lacked Keywords Plus, cited reference lists, and standardized affiliation data, which limited the scope of analysis that could be derived from this database.

Author names were disambiguated, institutional affiliations were standardized, and keyword synonyms and spelling variants were manually curated and merged within each dataset. Preprocessing was partially automated using Python 3.12, and all automated merges were verified by manual review (32).

### 2.4 Parallel bibliometric analysis

Because of the inherent differences in data completeness between the two databases, a parallel analysis strategy was adopted. The WoSCC dataset supported the full spectrum of bibliometric analyses, including citation-based metrics, co-citation networks, burst detection, and thematic evolution. The PubMed dataset, due to its field limitations, was primarily used to examine publication trends and keyword-based thematic patterns. This dual-database parallel approach allowed cross-validation of annual output trends and keyword co-occurrence results while acknowledging the distinct analytical strengths of each source.

#### 2.4.1 WoSCC-based analysis

VOSviewer (version 1.6.20) was used to construct co-authorship, co-citation, and keyword co-occurrence networks. CiteSpace (version 6.3.R3) was used for burst keyword detection and dual-map overlay generation. Bibliometrix (version 4.3.0, R package) was used for publication trend analysis, thematic mapping, testing of Lotka's and Bradford's laws. Scimago Graphica (version 1.0.53) was used for geographic collaboration visualization. Microsoft Excel 2024 was used for descriptive statistics.

#### 2.4.2 PubMed-based analysis

Given the absence of cited reference and Keywords Plus data, PubMed-derived analyses were limited to annual publication trends and author keyword co-occurrence patterns. Keyword frequency distributions and co-occurrence networks were generated using Bibliometrix. These results were compared with the corresponding WoSCC findings to assess the consistency of observed thematic patterns across databases.

#### 2.4.3 Citation comparison between document types

To compare the citation impact of original research articles vs. reviews, violin plots were generated to visualize the distribution of per-publication citation counts and numbers of cited references for each document type. Statistical significance was assessed using the Mann–Whitney *U* test, with *P* < 0.05 considered significant.

## 3 Results

### 3.1 Evolution of publication output and citation impact

The annual publication output of programmable mRNA-based therapeutics for myocardial infarction in the Web of Science Core Collection (WoSCC) database from 2012 to 2023 is illustrated in Figure 2A. Overall, the field demonstrated a fluctuating but overall upward trajectory, with annual publications increasing from approximately 40 articles in 2012 to nearly 90 articles in 2023, corresponding to an average annual growth rate of approximately 8%. Based on the temporal characteristics of publication growth, the research evolution of this field could be categorized into three distinct phases. The emerging phase (2012–2016) was characterized by relatively limited research activity, with annual publications remaining below 60. During the developmental phase (2017–2021), publication output increased steadily, accompanied by an average annual growth rate of approximately 10%. The accelerated expansion phase (2022 onward) was marked by a rapid increase in scientific output, with the number of publications reaching a peak of nearly 90 articles in 2023, indicating intensified research interest and accelerated development within this emerging field.

**Figure 2:**
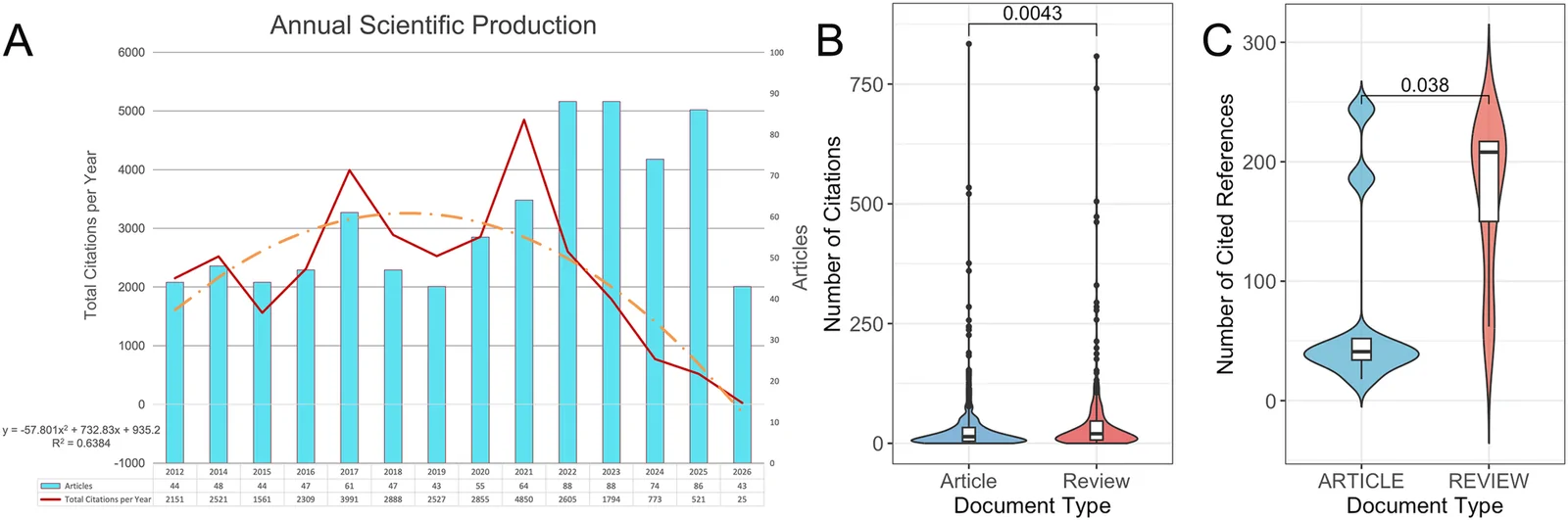
Analysis of papers published between 2012 and 2026. **(A)** Annual publication trends, cumulative citation counts, and polynomial fit curves for papers on the application of programmable mRNA in the treatment of myocardial infarction. **(B)** Violin plot of citation frequencies for articles and reviews. **(C)** Violin plot of the number of references for articles and reviews.

The citation frequency distributions of articles and reviews exhibited highly right-skewed distributions (Figure 2B). The majority of publications received relatively low-to-moderate citation counts, whereas a small proportion of highly cited studies accumulated more than 800 citations, suggesting that the overall citation impact of this field was largely driven by a limited number of influential publications. Statistical analysis revealed a significant difference in citation distributions between articles and reviews (*P* = 0.0043), indicating distinct citation patterns between original research articles and review publications.

Regarding reference citation volume (Figure 2C), review articles contained substantially more references than original articles, with a median of approximately 200 references per review compared with approximately 30–40 references per original research article. This difference was statistically significant (*P* = 0.038). These findings are consistent with the inherent characteristics of review articles, which typically integrate and synthesize a broad spectrum of existing evidence to provide comprehensive overviews of rapidly evolving research fields.

### 3.2 Global research landscape and international collaboration networks

At the geographical level, Figure 3A illustrates the global distribution of publications and the relative contributions of single-country publications (SCP) and multiple-country publications (MCP) across different countries. China and the United States constituted the leading contributors in the field, representing the first tier of global research number of publications (NP) (Table 1). China (NP = 270) ranked first in terms of publication output, followed by the United States (NP = 180), with both countries substantially surpassing other nations and serving as the primary drivers of scientific advancement in programmable mRNA-based therapeutics for myocardial infarction. Notably, both countries demonstrated a predominance of SCP, indicating strong domestic research capacity. In contrast, European countries, including Germany, Italy, the United Kingdom, and France, exhibited markedly higher MCP proportions, reflecting stronger tendencies toward international research collaboration.

**Figure 3:**
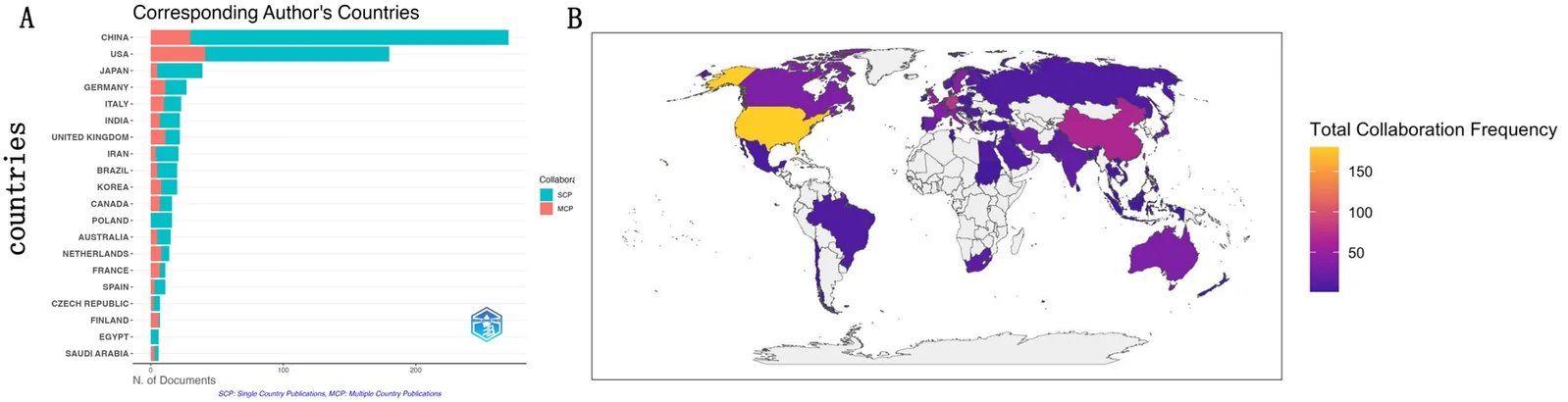
National and regional collaboration analysis. **(A)** Distribution of the top 20 countries/regions by number of publications in this field and types of collaboration. **(B)** Global collaboration heat map of countries/regions based on total collaboration frequency.

**Table 1:** Top 10 most productive countries and regions of corresponding authors.

Rank	Country	NP	NP %	SCP	MCP	MCP %
1	CHINA	270	32.5	240	30	11.1
2	USA	180	21.6	139	41	22.8
3	JAPAN	39	4.7	34	5	12.8
4	GERMANY	27	3.2	16	11	40.7
5	ITALY	23	2.7	13	10	43.5
6	INDIA	22	2.6	15	7	31.8
7	UNITED KINGDOM	22	2.6	11	11	50
8	IRAN	21	2.5	17	4	19
9	BRAZIL	20	2.4	15	5	25
10	KOREA	20	2.4	12	8	40

NP, number of publications; SCP, single-country publications; MCP, multiple-country publications (international collaborations); MCP ratio, MCP/NP.

The global collaboration intensity map further revealed distinct spatial patterns within the international cooperation network (Figure 3B). European countries and China showed high overall collaboration frequencies, emerging as the most active hubs within the global research network. North America countries, Japan, and Australia demonstrated moderate-to-high levels of international connectivity. Conversely, regions including South America, the Middle East, and South Asia exhibited relatively limited collaborative activity, while most African countries showed minimal participation in international research collaborations within this field.

At the institutional level, the collaboration network analysis revealed the distribution patterns of research productivity and interinstitutional cooperation within the field (Figure 4). Icahn School of Medicine at Mount Sinai (NP = 21) has the highest number of publications (Table 2). Harvard Medical School (TC = 2,982; TLS = 84) emerged as the most active institution, demonstrating the highest network connectivity. Other major contributors included the Chinese Academy of Medical Sciences (NP = 15), Shanghai Jiao Tong University (NP = 15), Shandong University (NP = 13), Fudan University (NP = 13), and Capital Medical University (NP = 13).

**Figure 4:**
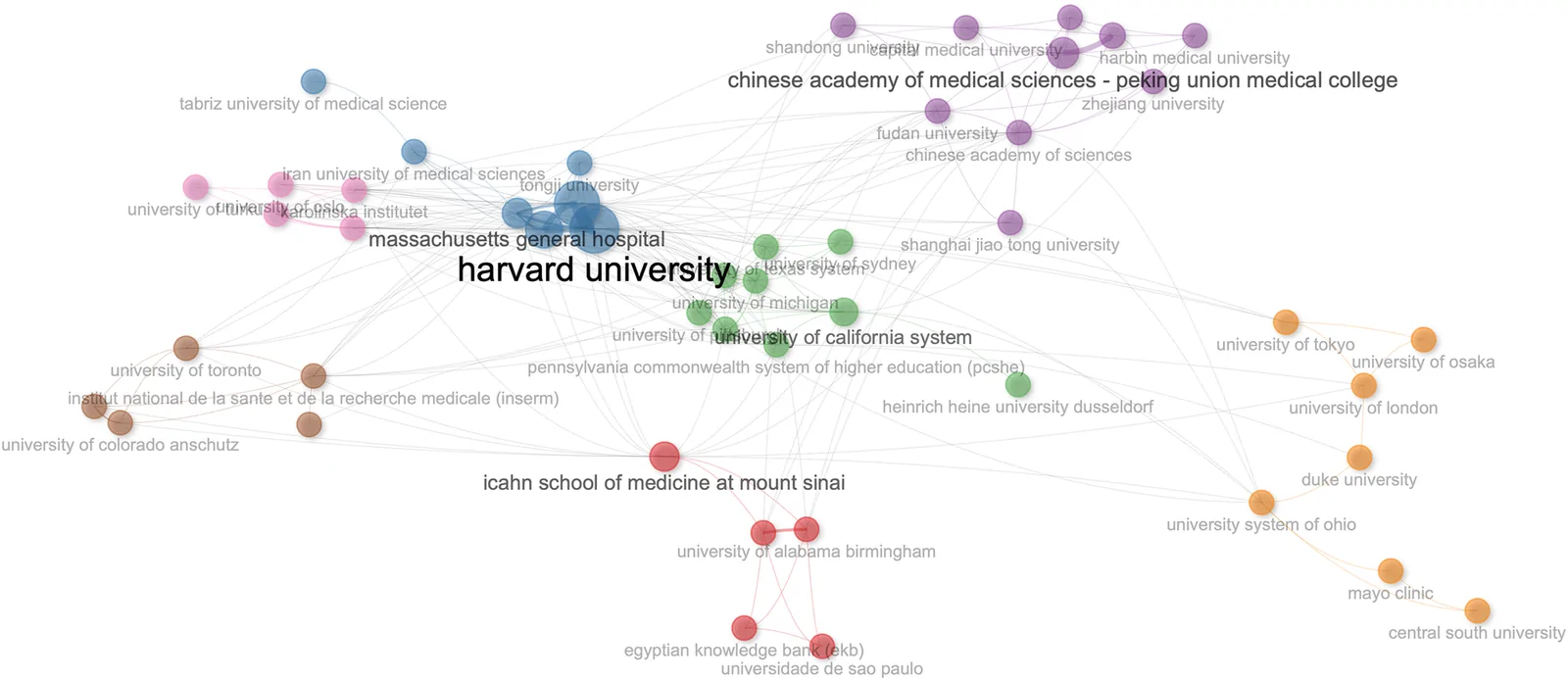
Network map of institutional partnerships.

**Table 2:** Top 10 most productive institutions.

Rank	Institution	NP	TC	TLS
1	icahn sch med mt sinai	21	1,730	46
2	harvard med sch	16	2,982	84
3	chinese acad med sci	15	469	44
4	shanghai jiao tong univ	15	433	18
5	shandong univ	13	973	23
6	fudan univ	13	394	45
7	capital med univ	13	277	18
8	karolinska inst	12	728	40
9	univ sao paulo	12	258	23
10	univ alabama birmingham	11	448	22

NP, number of publications; TC, total citations; TLS, total link strength.

The institutional collaboration network demonstrated the coexistence of regional clustering and extensive cross-regional partnerships. Institutions from China, Europe, and North America established dense internal collaborative structures, while maintaining frequent interregional interactions. These findings suggest that research on programmable mRNA-based therapeutics for myocardial infarction is characterized by both geographically concentrated innovation hubs and an increasingly interconnected global collaboration framework.

### 3.3 Journal landscape and publication characteristics

Among the top 10 journals ranked by publication output, *International Journal of Molecular Sciences* contributed the largest number of publications, followed by *Frontiers in Cardiovascular Medicine* and *Journal of Molecular and Cellular Cardiology* (Figure 5A). These journals represented major publication platforms for disseminating research findings related to programmable mRNA-based therapeutics in myocardial infarction.

**Figure 5:**
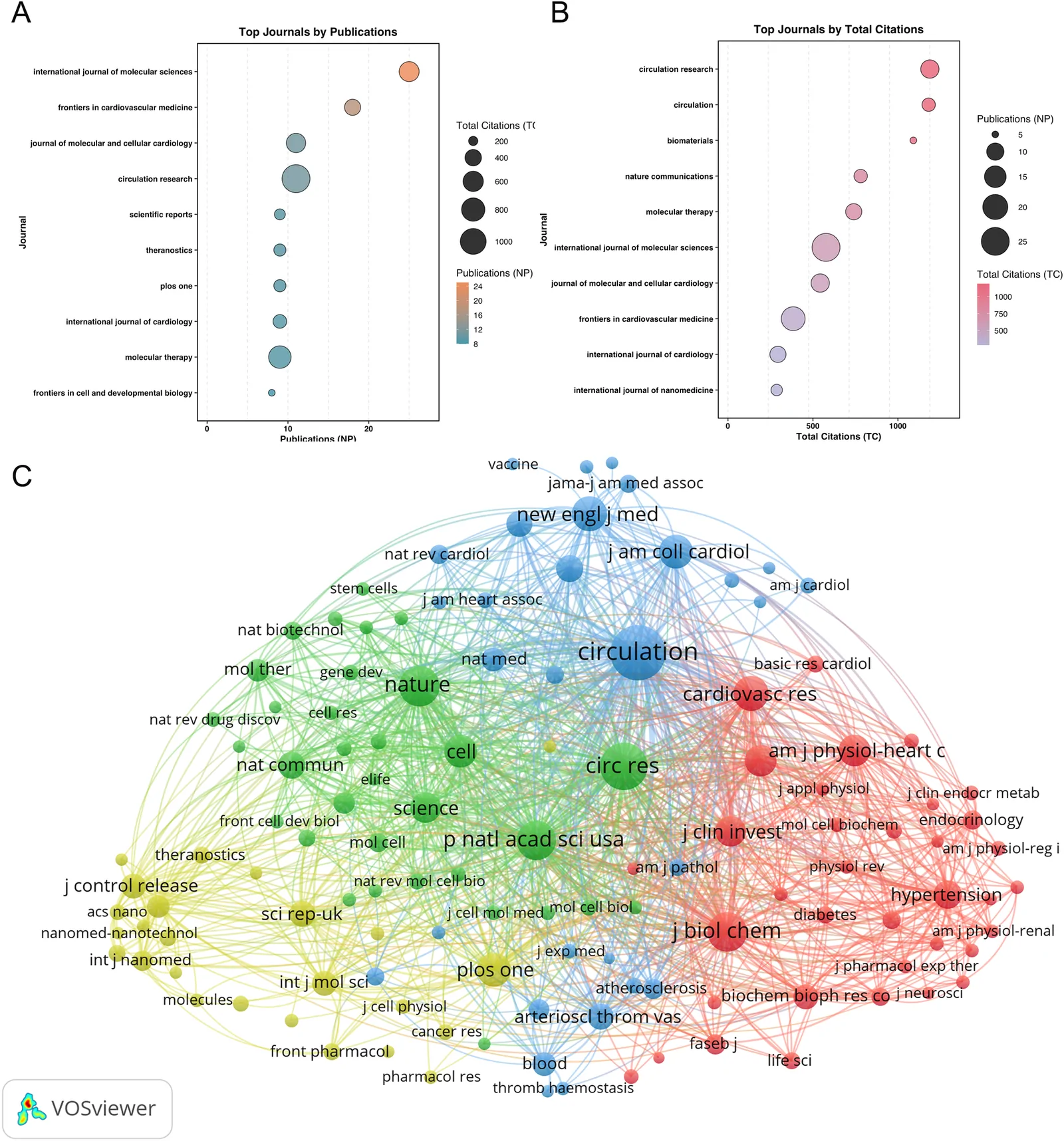
Journal output, citation impact, and co-citation network. **(A)** Top productive journals. **(B)** Top cited journals. **(C)** Node visualization of the journal co-citation network.

The journal co-citation analysis further identified the key knowledge sources and intellectual foundations underlying this research field (Figure 5B). *Circulation Research*, *Circulation*, and *Biomaterials* ranked as the three most frequently co-cited journals, highlighting their central roles in shaping the conceptual and methodological development of the field. Furthermore, the co-citation network revealed distinct knowledge structures and disciplinary connections, forming four major clusters centered on representative journals, including *Circulation*, *Journal of Biological Chemistry*, *Nature*, and *Journal of Controlled Release* (Figure 5C). These clusters reflected the interdisciplinary integration of cardiovascular biology, molecular mechanisms, biomaterials-based delivery systems, and advanced therapeutic technologies in the evolution of programmable mRNA research.

### 3.4 Author contributions and collaboration networks

At the author level, ZANGI LIOR (NP = 20) demonstrated the highest publication productivity, followed by MAGADUM, AJIT (NP = 12) and CHEPURKO, ELENA (NP = 11), who were among the principal contributors to the early development of the field of programmable mRNA-based therapeutic research (Table 3). Regarding scientific impact, ZANGI, LIOR also achieved the highest total citation count, with publications receiving consistently high annual citation rates during 2022–2024 (Figure 6A), highlighting his sustained influence within this research field.

**Table 3:** Top 10 most productive authors.

Rank	Author	NP	TC	H-index
1	ZANGI LIOR	20	1,260	15
2	MAGADUM AJIT	12	846	9
3	CHEPURKO ELENA	11	1,285	9
4	CHIEN KENNETH R.	8	587	8
5	HADAS YOAV	7	447	6
6	KURIAN ANN ANU	7	550	6
7	LI WEI	7	203	6
8	WANG WEI	7	109	4
9	HAJJAR ROGER J.	6	1,034	6
10	SZCZEPANSKA-SADOWSKA EWA	6	92	6

NP, number of publications; TC, total citations.

**Figure 6:**
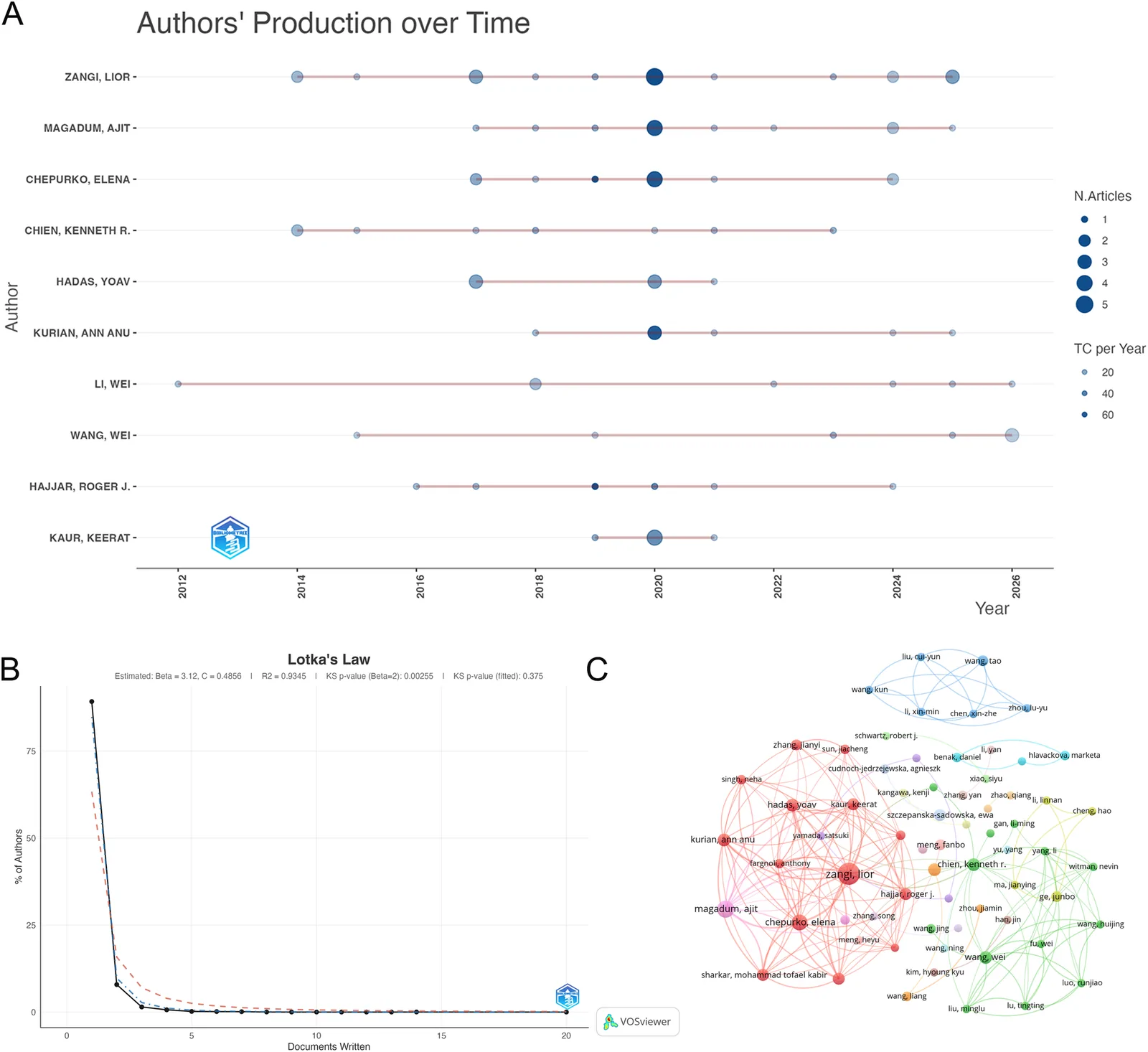
Author productivity and collaboration patterns. **(A)** Annual publication activity of the top 10 most productive authors. **(B)** Lotka's law distribution of author productivity. **(C)** Co-authorship network of authors.

Lotka's law analysis (Figure 6B) revealed a highly concentrated pattern of scientific productivity. Nearly 90% of authors contributed only one publication, indicating that knowledge production in this field is primarily driven by a relatively small number of highly productive research groups, whereas the majority of authors participated sporadically.

The author co-authorship network analysis (Figure 6C) identified four major collaborative clusters. The largest cluster was centered around ZANGi, LIOR (red cluster), followed by research groups led by WANG, WEIMIN (green cluster), CHEN, ZHE (blue cluster), and JIANG, PING (yellow cluster). Multiple intercluster connections were observed among these collaborative groups, suggesting the establishment of an increasingly interconnected research community with active cross-team scientific exchange.

### 3.5 Citation bursts and evolution of research frontiers

Citation burst analysis of references within the field of programmable mRNA-based therapeutics for myocardial infarction revealed a distinct evolutionary trajectory comprising three major phases: conceptual foundation and technological validation, accumulation of platform-level evidence and paradigm transition, and optimization of delivery systems for *in vivo* applications (Figure 7).

**Figure 7:**
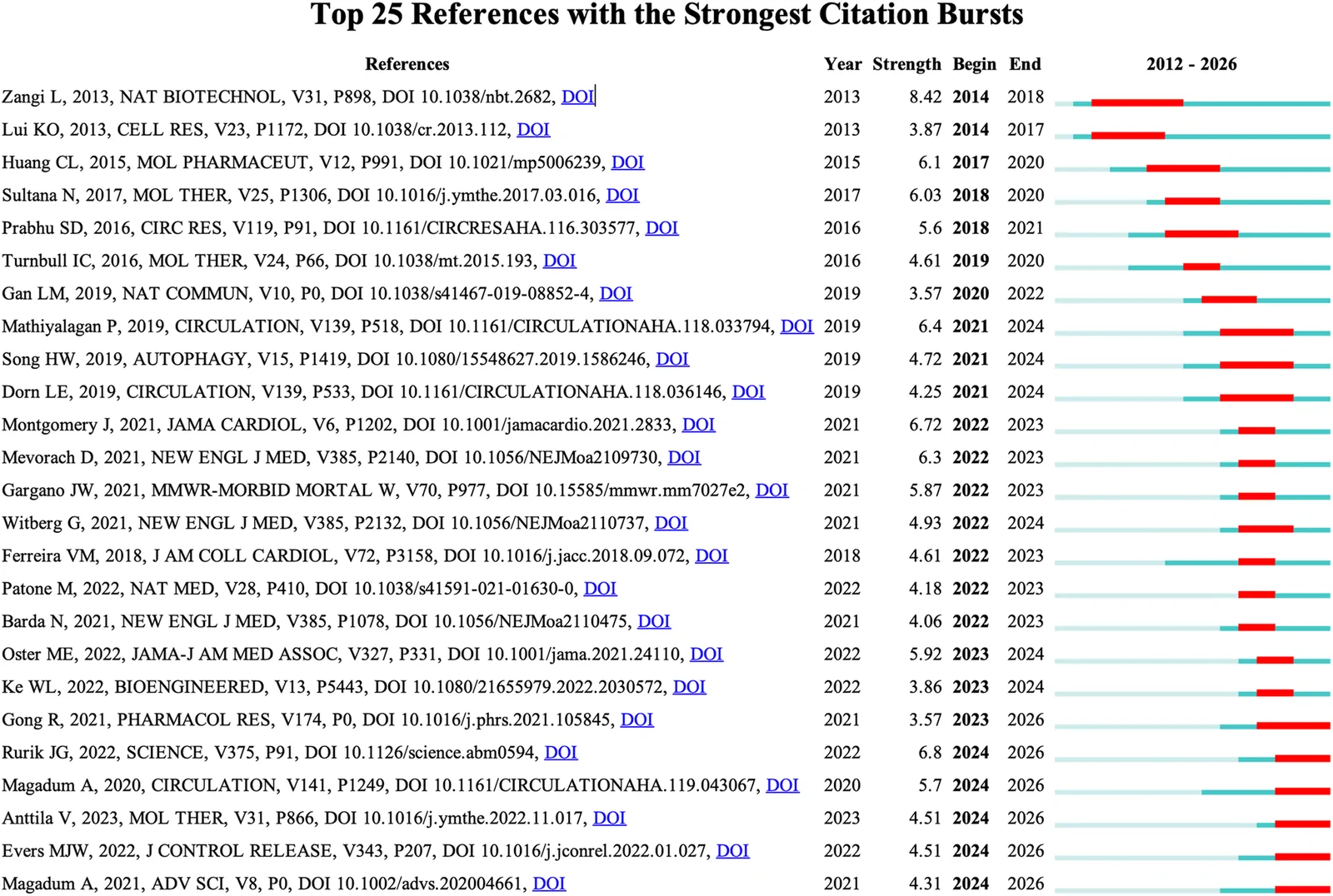
Citation burst analysis of the top 25 references.

During the first phase (2013–2017), the field established its conceptual foundation and mechanistic framework. The study by Zangi et al., published in *Nature Biotechnology* in 2013, exhibited the highest citation burst strength (8.42). This landmark study provided the first demonstration of the therapeutic potential of modified mRNA for cardiac repair *in vivo*, representing a critical milestone in the establishment of programmable mRNA-based approaches for myocardial regeneration. Concurrent studies during this period primarily focused on fundamental mechanisms, including cardiac regeneration, stem cell reprogramming, and mRNA modification technologies, thereby providing the theoretical and technical foundation for subsequent translational applications.

The second phase (2018–2022) was characterized by substantial platform-level expansion and accelerated translational advancement. A large number of highly cited publications with strong citation bursts emerged around 2021; however, many were not directly related to myocardial infarction but instead focused on the clinical development and safety evaluation of mRNA-based Coronavirus Disease 2019 (COVID-19) vaccines. This cross-disciplinary spillover effect highlighted the broader validation of mRNA platforms in terms of safety, scalability, and therapeutic feasibility, substantially accelerating research momentum in cardiovascular applications. Notably, the study by Rurik et al., published in *Science*, demonstrated a major therapeutic paradigm shift by enabling *in vivo* mRNA-mediated reprogramming of T cells into chimeric antigen receptor T (CAR-T) cells for the treatment of cardiac injury, achieving a high citation burst strength (6.8) and emerging as one of the most influential studies during this phase.

The third phase (2023–2026) has been primarily driven by advances in precision delivery technologies. Citation bursts persisted during 2024–2026, with the study by Anttila et al., published in *Molecular Therapy* in 2023, highlighting the development and optimization of mRNA delivery platforms as a central research direction. These findings indicate a progressive transition of the field from proof-of-concept validation toward engineering-based strategies aimed at improving tissue specificity, delivery efficiency, and therapeutic applicability.

### 3.6 Keyword co-occurrence networks and thematic evolution

The keyword co-occurrence network (Figure 8A) revealed four major thematic clusters within the field. These included oxidative stress-related mechanisms (green cluster), pathological mechanisms of myocardial infarction involving inflammation and nuclear factor kappa-light-chain-enhancer of activated B cells (NF-κB) signaling (purple cluster), cardiovascular diseases and therapeutic delivery systems (blue cluster), and mRNA expression regulation and target gene functions (red cluster). Collectively, these clusters highlighted the multidisciplinary integration of molecular mechanisms, disease pathology, gene regulation, and delivery technologies in the development of programmable mRNA-based therapeutics for myocardial infarction.

**Figure 8:**
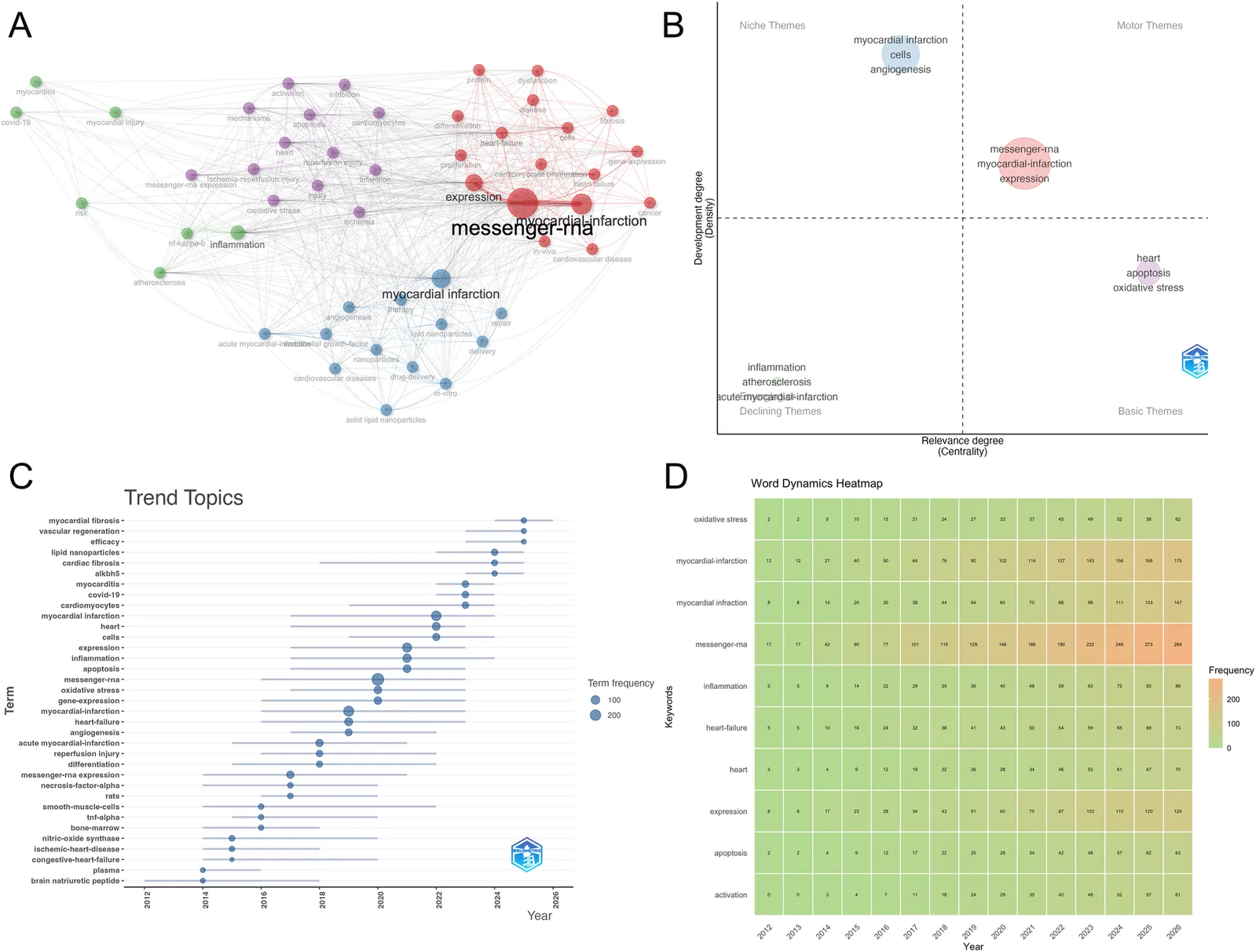
Keyword analysis of the primary domain and cross-analysis of sub-themes. **(A)** Node visualization of the keyword co-occurrence network in the primary domain. **(B)** research topic overview chart **(C)** Chronological timeline of thematic trend distribution in the primary domain. **(D)** Density heatmap of keywords in the primary domain.

The strategic diagram analysis (Figure 8B) further characterized the maturity and centrality of different research themes. The terms “messenger RNA,” “myocardial infarction,” and “expression” were located within the core motor themes, exhibiting both high density and high centrality (Table 4). These findings indicate that mRNA-mediated regulation of target gene expression has become the central conceptual framework driving this research field. In contrast, themes such as “angiogenesis” demonstrated relatively high development density but limited centrality, suggesting promising yet insufficiently integrated research directions. Meanwhile, fundamental biological themes, including “apoptosis” and “oxidative stress,” exhibited high centrality but comparatively lower developmental momentum, reflecting their established importance but slower thematic expansion.

**Table 4:** Top 20 most frequent keywords.

Rank	Words	Occurrences
1	messenger-rna	284
2	myocardial-infarction	175
3	myocardial infarction	147
4	expression	124
5	inflammation	89
6	heart-failure	73
7	heart	70
8	apoptosis	63
9	oxidative stress	62
10	activation	61
11	gene-expression	60
12	atherosclerosis	58
13	acute myocardial-infarction	53
14	heart failure	52
15	messenger-rna expression	51
16	cells	46
17	angiogenesis	43
18	*in-vitro*	41
19	solid lipid nanoparticles	38
20	*in-vivo*	37

Thematic evolution analysis (Figure 8C) revealed a progressive transition of research priorities across three distinct periods. During 2012–2016, research primarily focused on clinical manifestations and classical molecular pathways, including heart failure-related indicators such as brain natriuretic peptide (BNP) and inflammatory signaling pathways represented by NF-κB. From 2016 to 2020, the research focus gradually shifted toward cellular intervention strategies involving apoptosis regulation, stem cell-based approaches, and gene expression modulation. Since 2020, emerging themes including myocardial fibrosis, vascular regeneration, lipid metabolism, and reversal of cardiac fibrosis have become sustained research hotspots, reflecting a transition toward regenerative and translational therapeutic strategies.

The dynamic thematic heatmap (Figure 8D) further confirmed this evolutionary shift. The occurrence frequencies of “messenger RNA” and “myocardial infarction” increased sharply after 2020, reaching their highest levels during 2022–2023. During the same period, pathological concepts including “inflammation” and “oxidative stress” also reached peak activity, indicating that recent investigations have increasingly focused on leveraging mRNA technologies to modulate post-infarction inflammatory responses and oxidative injury. Furthermore, the continued prominence of themes such as “myocardial fibrosis” and “angiogenesis” suggests that fibrosis reversal and vascular regeneration are becoming prominent frontiers in the future development of programmable mRNA-based therapies for myocardial infarction.

### 3.7 PubMed-based research hotspots and thematic evolution

The annual publication output derived from the PubMed database (Figure 9A) demonstrated a relatively stable growth pattern between 2012 and 2025, with annual publications ranging from 252 to 330 articles. The research output reached a local peak in 2025, with 323 publications, indicating sustained scientific interest in mRNA-related investigations of myocardial infarction.

**Figure 9:**
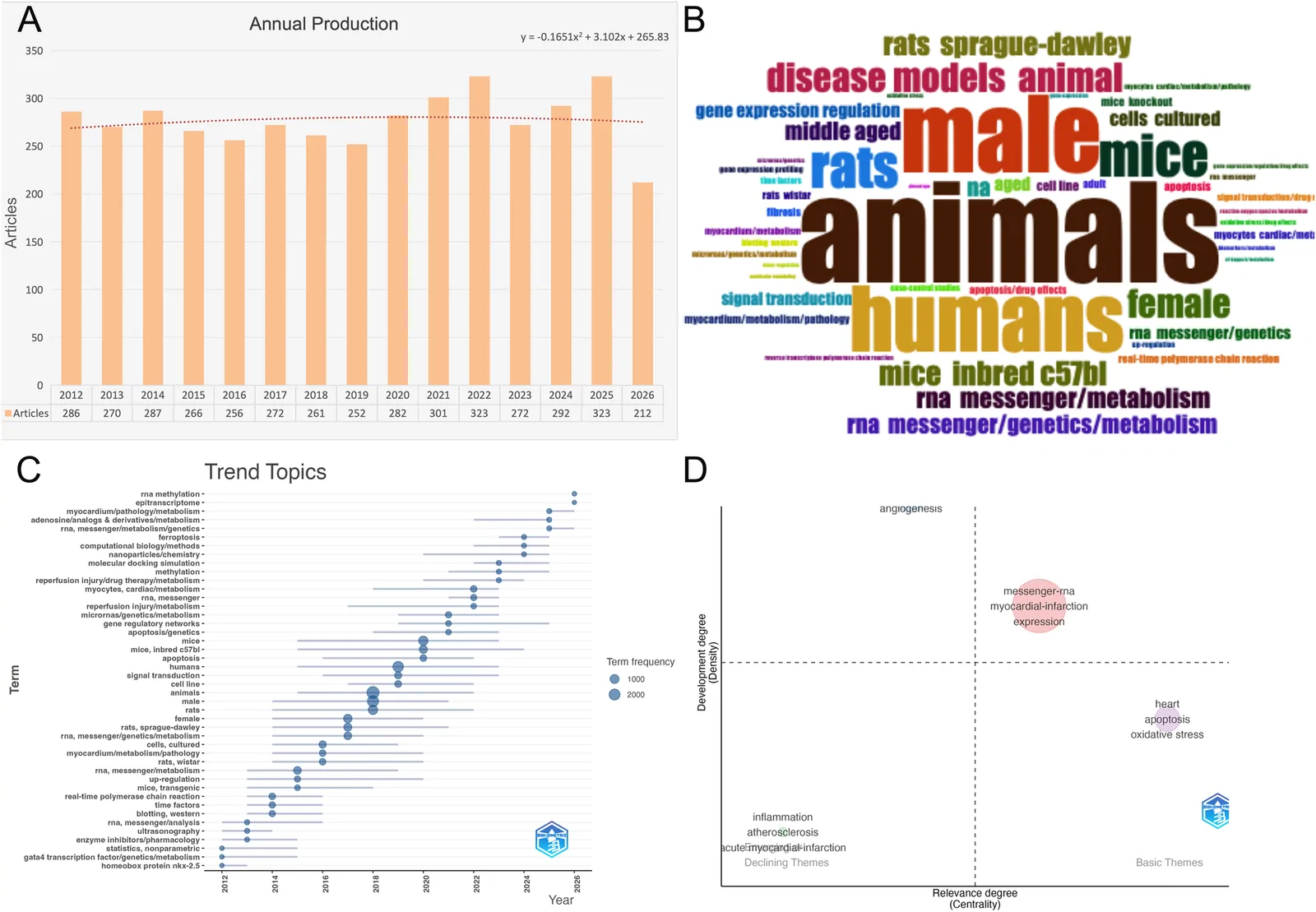
Based on the PubMed database, this analysis examines annual output trends, characteristics of high-frequency research, the evolution of hot topics, and strategic positioning of themes in this field. **(A)** Bar chart showing annual publication volume and a second-order polynomial trend fit. **(B)** Word cloud of high-frequency research subjects, experimental models, and core terms from the included literature. **(C)** Trend chart illustrating the dynamic evolution of core research themes and high-frequency keywords over time. **(D)** Strategic coordinate diagram classifying core driving themes, foundational themes, and peripheral themes based on centrality and development.

The high-frequency keyword analysis (Figure 9B) revealed a strong preclinical research orientation within this field. Experimental terms, including “Animals,” “Rats,” “Mice,” and “Rats, Sprague-Dawley,” dominated the keyword landscape, suggesting that a substantial proportion of studies remain focused on animal models and cellular experimental systems. The frequent occurrence of “Male” and “Female” indicated increasing attention toward sex-related biological differences, whereas mechanistic terms such as “signal transduction” and “gene expression regulation” represented the central framework of fundamental investigations.

The historical evolution of research themes (Figure 9C) delineated a progressive three-stage developmental trajectory. During the early phase (2012–2016), research primarily concentrated on signal transduction pathways, gene expression regulation, and fundamental pathological mechanisms associated with mRNA biology. The intermediate phase (2017–2021) witnessed a transition toward microRNA regulation, apoptosis, fibrosis, and ischemia–reperfusion injury, reflecting increasing exploration of non-coding RNA-mediated interventions and molecular strategies for myocardial repair. Since 2022, the research landscape has shifted toward RNA methylation and immunotherapeutic approaches, marking the emergence of a more advanced research paradigm centered on epitranscriptomic regulation, including N^6^-methyladenosine (m^6^A) modification, and RNA-mediated immune modulation.

The strategic diagram analysis (Figure 9D) further confirmed the central position of “messenger RNA” and “myocardial infarction” as core motor themes located within the high-centrality and high-development quadrant. In contrast, “angiogenesis” emerged as a promising but relatively underdeveloped theme, indicating a valuable direction for future exploration in programmable mRNA-based cardiac regenerative therapies.

## 4 Discussion

In this study, we performed a comprehensive bibliometric analysis of the rapidly evolving field of programmable mRNA-based therapeutics for myocardial infarction and identified several major findings. First, this research area has entered a phase of accelerated expansion since 2022, characterized by a substantial increase in publication output. The platform-level spillover effect from COVID-19 mRNA vaccine development, together with transformative technological advances such as *in vivo* CAR-T cells reprogramming, may have contributed substantially to this growth behind this rapid growth (33). Second, the research landscape has undergone a pronounced thematic transition, progressing from early investigations of signal transduction and apoptotic mechanisms, through microRNA- and stem cell-based interventions, toward emerging frontiers including myocardial fibrosis reversal, angiogenesis, RNA methylation regulation, and immunotherapeutic strategies (34). Third, although China and the United States have dominated global publication productivity, European countries have demonstrated stronger international collaborative intensity, revealing a global research landscape characterized by concentrated scientific output but uneven collaboration patterns. Collectively, these findings provide a comprehensive overview of the knowledge evolution and emerging directions in programmable mRNA-based cardiac therapeutics, which are discussed in detail below.

Our analysis of keyword bursts and thematic evolution suggested a paradigm shift in this field, transitioning from “passive cardioprotection” toward “active myocardial regeneration.” During the early phase (2012–2016), research activities were primarily focused on classical pathological mechanisms and clinical indicators, including NF-κB signaling, signal transduction pathways, and heart failure-related parameters, reflecting an initial stage of knowledge accumulation centered on disease characterization and mechanistic understanding.

During the intermediate phase (2017–2021), research priorities gradually shifted toward microRNA regulation, apoptosis modulation, stem cell-based approaches, and gene expression control, suggesting that investigators began to explore active manipulation of cardiomyocyte fate through RNA-based interventions and molecular reprogramming strategies. In the most recent phase (2022–2026), emerging themes including myocardial fibrosis, angiogenesis, lipid metabolism, and RNA methylation regulation have become sustained research priorities, indicating a further transition toward regenerative and precision therapeutic strategies.

This evolutionary trajectory is consistent with the pioneering work of Zangi et al., which demonstrated the potential of modified mRNA for promoting cardiac regeneration, and aligns with the broader transformation of mRNA technologies from vaccine platforms toward regenerative medicine applications (35). Several factors may explain this transition. First, advances in chemical modification technologies and lipid nanoparticle (LNP)-based delivery systems have substantially improved the feasibility of *in vivo* mRNA administration (36). Second, the large-scale clinical validation of mRNA platforms during the COVID-19 pandemic has provided unprecedented evidence regarding their safety, scalability, and therapeutic potential, thereby accelerating the expansion of mRNA technologies into cardiovascular applications.

Collectively, these findings suggest that the conceptual framework of this field has evolved from understanding disease mechanisms toward actively intervening in disease progression, with increasing emphasis on the precise modulation of the regenerative microenvironment. Future research directions should prioritize integrated therapeutic strategies combining fibrosis reversal and angiogenesis enhancement, which may represent promising approaches for improving the translational potential of mRNA-based cardiac regenerative therapies.

Our analysis identified angiogenesis as a distinctive thematic domain positioned within the quadrant of high density but relatively low centrality in the strategic diagram, suggesting that it represents a promising yet incompletely integrated research direction suggesting that the theme remains relatively specialized and insufficiently integrated into the broader field (37). This finding is partially consistent with previous bibliometric studies in the field of cardiovascular regeneration, in which angiogenesis has been recognized as a critical therapeutic target for cardiac repair (38). However, our analysis further indicates that angiogenesis currently resides at a transitional stage between an emerging niche and a mainstream research focus, rather than being fully incorporated into the central knowledge structure of the field.

Several factors may explain this phenomenon. First, the delivery of proangiogenic mRNA therapeutics continues to face challenges due to limited protein translation efficiency within the hypoxic microenvironment of infarcted myocardium (39). Second, single-modality angiogenic therapies have demonstrated insufficient translation into meaningful improvements in cardiac function, particularly in large animal models, highlighting the limitations of angiogenesis-based strategies alone (40).

A plausible interpretation of these findings is that angiogenesis in isolation may not be sufficient to achieve functional myocardial regeneration without simultaneous regulation of cardiomyocyte survival and fibrotic remodeling (41). Therefore, future research should move beyond single-target angiogenic approaches and focus on multifactorial synergistic mRNA delivery strategies integrating angiogenesis promotion, antifibrotic regulation, and myocardial protection (42). Such combination-based therapeutic designs may provide a critical pathway for improving the clinical translatability of mRNA-based cardiac regenerative therapies.

Our analysis further revealed that RNA methylation, particularly N^6^-methyladenosine (m^6^A) modification, and immunotherapy-related themes emerged as high-intensity citation burst keywords after 2022, indicating that the field has entered a new developmental stage increasingly centered on epitranscriptomic regulation and RNA-mediated immune modulation. This observation is highly consistent with the growing recognition of epitranscriptomics as a critical regulatory layer across modern biomedical research (43).

Recent studies have demonstrated that m^6^A modification plays essential roles in maintaining cardiomyocyte homeostasis and regulating cardiac remodeling following myocardial injury (44, 45). Meanwhile, the emergence of immunotherapy-related research themes appears to be closely associated with transformative advances in RNA-based immune engineering, particularly the development of *in vivo* CAR-T cells reprogramming strategies for cardiac repair (46).

A plausible explanation for this emerging trend is that the unique programmability of mRNA platforms enables precise and transient regulation of therapeutic protein expression while simultaneously providing opportunities to modulate broader regulatory networks, including epitranscriptomic states (47). As the biological functions of RNA modifications become increasingly understood, programmable mRNA technologies may evolve beyond conventional protein replacement approaches toward more sophisticated regulatory therapeutics (48).

Collectively, these findings suggest that epitranscriptomic-based interventions may represent the next frontier of programmable mRNA-mediated cardiac regeneration, providing new technological dimensions for advancing precision cardiac repair and expanding the therapeutic landscape of mRNA-based medicine.

Compared with conventional narrative reviews, this study employed a systematic, quantitative, and visualization-based framework to objectively map the global knowledge landscape of programmable mRNA-based therapeutics for myocardial infarction. Through comprehensive bibliometric analyses, we identified key contributors, including Zangi and other influential researchers, together with their landmark studies that established the foundation of this field. Furthermore, our approach enabled the detection of emerging research frontiers, including angiogenesis and epitranscriptomic regulation, which may represent future directions for therapeutic development.

From a translational perspective, the observed shift in research priorities—from fundamental mechanistic investigations toward precision modulation of inflammation, fibrosis reversal, and vascular regeneration—provides quantitative evidence to guide the selection of promising entry points for future clinical research. In addition, the analysis of global collaboration patterns and regional disparities may offer valuable insights for optimizing the design and distribution of international clinical trial networks.

Beyond the specific application of mRNA-based cardiac therapeutics, the methodological framework established in this study provides a scalable and generalizable strategy for mapping knowledge structures, identifying emerging trends, and guiding translational priorities across other rapidly evolving biomedical fields.

Several limitations of this study should be acknowledged. First, although the Web of Science Core Collection (WoSCC) and PubMed databases represent authoritative and widely used sources for biomedical literature retrieval, relevant studies indexed in other databases, such as Embase, Scopus, and Google Scholar, may not have been captured. Therefore, the possibility of incomplete literature coverage cannot be entirely excluded.

Second, language bias may exist because this study included only English-language publications. Consequently, important research findings published in other languages may have been underrepresented, particularly locally conducted studies from highly active non-English-speaking countries, including China and Germany.

Third, citation-based analyses are inherently influenced by temporal factors. High-quality studies published between 2024 and 2026 may have been underestimated due to insufficient citation accumulation time, and the long-term impact of recently emerging research trends identified through citation bursts requires further validation with extended follow-up periods.

Fourth, the establishment of minimum frequency thresholds for keyword co-occurrence analysis involves a degree of methodological subjectivity. Different threshold settings may potentially influence the resulting network structures, clustering patterns, and interpretation of thematic relationships.

Finally, this study was limited to original research articles and reviews, while other forms of scientific communication, including conference proceedings and preprints, were not included. Therefore, some emerging advances and rapidly evolving developments in the field may not have been fully captured.

## 5 Conclusion

By integrating bibliometric analyses from the Web of Science Core Collection (WoSCC) and PubMed databases, this study systematically characterized the global research landscape and evolutionary trajectory of programmable mRNA-based therapeutics for myocardial infarction from 2012 to 2026. Our findings demonstrate that, following a period of gradual accumulation during the early developmental stages, this field entered a phase of rapid expansion after 2022. The technological validation and accelerated maturation of mRNA platforms during the COVID-19 pandemic may have been an important contributor of this accelerated growth. The thematic landscape has undergone a profound transition, evolving from early investigations focused on fundamental mechanisms such as signal transduction and apoptosis, through an intermediate stage involving microRNA regulation and stem cell-based interventions, toward emerging frontiers including myocardial fibrosis reversal, angiogenesis, RNA methylation, particularly m^6^A modification, and immunotherapy. This progression reflects a transition from cardioprotection-oriented strategies toward regenerative approaches and, more recently, precision modulation of the regenerative microenvironment.

China and the United States currently represent the leading contributors in terms of publication productivity; however, European countries showed higher proportions of multiple-country publications and more intensive international collaboration of international collaboration, whereas participation from low- and middle-income regions remains comparatively limited. A small number of highly influential researchers, exemplified by Zangi, together with their landmark contributions, constitute the intellectual foundation of this field. Meanwhile, the observed Lotka's law distribution, in which nearly 90% of authors contributed only one publication, indicates that the field has not yet developed a mature and widely distributed research community.

Looking forward, several priorities are likely to determine the future translational success of programmable mRNA-based cardiac therapeutics. These include the development of cardiac-targeted delivery systems with improved specificity, efficiency, and reduced immunogenicity, exploration of epitranscriptomic regulatory strategies represented by m^6^A modification, and the establishment of multifunctional therapeutic platforms integrating angiogenesis promotion, antifibrotic regulation, and cardiomyocyte protection. Collectively, these advances may provide critical pathways for translating programmable mRNA technologies from experimental research toward clinical application.

This study provides a comprehensive quantitative framework for understanding the evolution, knowledge structure, and emerging priorities of programmable mRNA-based therapies for myocardial infarction, offering evidence-based guidance for future collaboration strategies, research planning.

## Data Availability

Publicly available datasets were analyzed in this study. The data were retrieved from the Web of Science Core Collection (WoSCC) and PubMed databases. The complete retrieval strategies, including search terms, Boolean operators, and the study period, are provided in the Methods section and Supplementary Table S1.
