# Supplementary material for: Global landscape and emerging trends in programmable mRNA-based therapeutics for myocardial infarction: a multi-database bibliometric analysis
Source: Front Med (Lausanne). 2026 Sep 28;13:1957977. doi: 10.3389/fmed.2026.1957977 (PMC13654849; doi:10.3389/fmed.2026.1957977)
Supplement: Supplementary file 1 [file Table1.docx]

**Supplementary Table 1. Search Strategies Employed in the Web of Science Core Collection (WoSCC) and PubMed Databases**

**Web of Science Core Collection (WoSCC)**

Field tag: Topic Search (TS). Time span: January 1, 2012, to July 15, 2026. The TS field searches titles, abstracts, author keywords, and Keywords Plus.

**Search strategy:**

TS=("modified mRNA" OR modRNA OR "messenger RNA" OR "mRNA therapy" OR "mRNA therapeutic" OR "mRNA-based" OR "mRNA therapeutics" OR "self-amplifying mRNA" OR saRNA OR "synthetic mRNA" OR "in vitro transcribed mRNA" OR "IVT mRNA" OR "mRNA vaccine" OR "mRNA deliver" OR "mRNA transfect" OR "mRNA encapsulat" OR "lipid nanoparticle" OR LNP) OR ("mRNA" OR "messenger RNA") NEAR/15 (therap OR deliver* OR nanoparticle* OR lipid* OR liposome* OR encapsulat* OR transfect* OR regeneration OR repair OR engineering) AND TS=("myocardial infarction" OR "acute myocardial infarction" OR "heart attack" OR "myocardial ischemia" OR "ischemic heart disease" OR "ischemic cardiomyopathy" OR "cardiac repair" OR "myocardial injury" OR "myocardial regeneration" OR "cardiac regeneration") NOT TS=(miRNA OR microRNA OR siRNA OR circRNA OR lncRNA)

**PubMed**

Field tag: TITLE-ABS-KEY. Time span: January 1, 2012, to July 15, 2026. The TITLE-ABS-KEY field searches titles, abstracts, and keyword fields indexed in Scopus. Its coverage is comparable to, but not identical with, the WoSCC Topic Search field.

**Search strategy:** TITLE-ABS-KEY(("Modified mRNA"[Title/Abstract] OR modRNA[Title/Abstract] OR "therapeutic mRNA"[Title/Abstract] OR "mRNA therapy"[Title/Abstract] OR "mRNA therapeutic*"[Title/Abstract] OR "mRNA-based therap*"[Title/Abstract] OR "synthetic mRNA"[Title/Abstract] OR "self-amplifying mRNA"[Title/Abstract] OR saRNA[Title/Abstract] OR "in vitro transcribed mRNA"[Title/Abstract] OR "IVT mRNA"[Title/Abstract] OR "engineered mRNA"[Title/Abstract] OR "mRNA deliver*"[Title/Abstract] OR "mRNA transfect*"[Title/Abstract] OR "mRNA encapsulat*"[Title/Abstract] OR "lipid nanoparticle*"[Title/Abstract] OR LNP*[Title/Abstract] OR (("mRNA"[Title/Abstract] OR "messenger RNA"[Title/Abstract]) AND (therap*[Title/Abstract] OR deliver*[Title/Abstract] OR nanoparticle*[Title/Abstract] OR lipid*[Title/Abstract] OR liposome*[Title/Abstract] OR encapsulat*[Title/Abstract] OR transfect*[Title/Abstract] OR repair*[Title/Abstract] OR regenerat*[Title/Abstract] OR engineer*[Title/Abstract])) OR "RNA, Messenger"[Mesh])) AND (("Myocardial Infarction"[Mesh] OR "Heart Failure"[Mesh] OR "Myocardial Ischemia"[Mesh] OR "myocardial infarction"[Title/Abstract] OR "acute myocardial infarction"[Title/Abstract] OR "heart attack"[Title/Abstract] OR "myocardial ischemia"[Title/Abstract] OR "cardiac ischemia"[Title/Abstract] OR "ischemic heart disease"[Title/Abstract] OR "ischemic cardiomyopathy"[Title/Abstract] OR "myocardial injury"[Title/Abstract] OR "cardiac injury"[Title/Abstract] OR "cardiac repair"[Title/Abstract] OR "cardiac regeneration"[Title/Abstract] OR "myocardial regeneration"[Title/Abstract] OR "ventricular remodeling"[Title/Abstract] OR "left ventricular remodeling"[Title/Abstract] OR "ischemia reperfusion injury"[Title/Abstract] OR "ischemia-reperfusion injury"[Title/Abstract] OR "post infarction"[Title/Abstract] OR "post-infarction"[Title/Abstract] OR "heart failure"[Title/Abstract]))) NOT (miRNA[Title] OR microRNA[Title] OR siRNA[Title] OR circRNA[Title] OR lncRNA[Title])) AND 2012/01/01:2026/07/15[dp]
